# Phenotypic and Genetic Heterogeneity in *Vibrio cholerae* O139 Isolated from Cholera Cases in Delhi, India during 2001–2006

**DOI:** 10.3389/fmicb.2016.01250

**Published:** 2016-08-09

**Authors:** Raikamal Ghosh, Naresh C. Sharma, Kalpataru Halder, Rupak K. Bhadra, Goutam Chowdhury, Gururaja P. Pazhani, Sumio Shinoda, Asish K. Mukhopadhyay, G. Balakrish Nair, Thadavarayan Ramamurthy

**Affiliations:** ^1^Division of Bacteriology, National Institute of Cholera and Enteric DiseasesKolkata, India; ^2^Maharishi Valmiki Infectious Diseases HospitalDelhi, India; ^3^Infectious Diseases and Immunology Division, Council of Scientific and Industrial Research-Indian Institute of Chemical BiologyKolkata, India; ^4^Collaborative Research Center of Okayama University for Infectious Diseases in India, National Institute of Cholera and Enteric DiseasesKolkata, India; ^5^Center for Human Microbial Ecology, Translational Health Science and Technology InstituteFaridabad, India

**Keywords:** *V. cholerae* O139, ribotypes, CT genotype, CTX prophage, PFGE

## Abstract

Incidence of epidemic *Vibrio cholerae* serogroup O139 has declined in cholera endemic countries. However, sporadic cholera caused by *V. cholerae* O139 with notable genetic changes is still reported from many regions. In the present study, 42 *V. cholerae* O139 strains isolated from 2001 to 2006 in Delhi, India, were retrospectively analyzed to understand their phenotype and molecular characteristics. The majority of isolates were resistant to ampicillin, furazolidone and nalidixic acid. Though the integrative conjugative element was detected in all the O139 isolates, the 2004–2006 isolates remained susceptible to co-trimoxazole, chloramphenicol, and streptomycin. Cholera toxin genotype 1 was present in the majority of the O139 isolates while few had type 3 or a novel type 4. In the cholera toxin encoding gene (*ctx*) restriction fragment length polymorphism, the majority of the isolates harbored three copies of CTX element, of which one was truncated. In this study, the *ctx* was detected for the first time in the small chromosome of *V. cholerae* O139 and one isolate harbored 5 copies of CTX element, of which 3 were truncated. The ribotype BII pattern was found in most of the O139 isolates. Three *V. cholerae* O139 isolated in 2001 had a new ribotype BVIII. Pulsed-field gel electrophoresis analysis revealed clonal variation in 2001 isolates compared to the 2004–2006 isolates. Molecular changes in *V. cholerae* O139 have to be closely monitored as this information may help in understanding the changing genetic features of this pathogen in relation to the epidemiology of cholera.

## Introduction

The aquatic bacterium *Vibrio cholerae* is the causative agent of cholera or cholera-like diarrhea in humans. Of the 206 serogroups identified in this species (Yamai et al., 1997), the serogroups O1 and O139 are responsible for global cholera epidemics. *V. cholerae* serogroup O1 is further divided into two biotypes, classical and El Tor and each has two distinct serotypes, Inaba and Ogawa. The classical biotype was associated with cholera in first six pandemics (Sack et al., 2004). The current 7th cholera pandemic is represented by *V. cholerae* O1 El Tor biotype, which became dominant from 1961 and gradually replaced the classical biotype from the global cholera scenario. *V. cholerae* O139 serogroup emerged in 1992 by replacing the El Tor biotype in the Indian subcontinent and spread to more than 14 countries in the following years (Nair et al., 1994a; Siddique et al., 1996; Ramamurthy et al., 2003). Emergence of *V. cholerae* O139 serogroup was thought to be the beginning of the 8th cholera pandemic considering the rapid spread of the pathogen (Nair et al., 1994b). However, after causing large cholera epidemics in 1993, the serogroup O139 disappeared abruptly from the endemic scenario ensuing resurgence of *V. cholerae* O1 El Tor biotype in cholera endemic regions (Sharma et al., 1997). Until late 1999, there has been periodic shift between El Tor and O139 in India and Bangladesh (Basu et al., 2000; Faruque et al., 2003a). In 2008, the incidence of *V. cholerae* O139 in China was 32% among cholera cases (WHO, 2009) and continued until 2012 (Zhang et al., 2014).

In *V. cholerae* O139, changes in the antimicrobial susceptibility patterns and arrangement of genetic elements, especially the organization of ribosomal RNA operons, location, and arrangement of cholera toxin prophages (CTXΦ) were reported during its emergence on several occasions (Sharma et al., 1997; Faruque et al., 2003a; Nandi et al., 2003; Chatterjee et al., 2007; Ghosh et al., 2008). Initial genetic analysis showed that emergence of *V. cholerae* O139 may be due to the insertion of a novel 35-kb *wbf* gene that encodes O139-somatic (O) antigen in a *V. cholerae* serogroup O22 strain or due to the loss of a 22-kb *wbe* region in a *V. cholerae* O1 that encodes the O1 antigen (Yamasaki et al., 1999). The whole genome sequence analysis by Chun et al. (2009) confirmed the above finding, i.e., substitution of the gene cluster coding for the O139 antigen took place by horizontal gene transfer but not the deletion.

Based on the amino acid changes, the B-subunits of CT have been designated into several CT-genotypes or *ctxB* alleles (Safa et al., 2008; Raychoudhuri et al., 2009). CT genotyping (*ctxB* allele) can be made using Mismatch amplification mutation assay (MAMA) PCR (Morita et al., 2008). CT genotype 1 is reported in strains of the classical biotype worldwide and in US Gulf Coast, genotype 2 is found in El Tor biotype strains from Australia, and genotype 3 is prevalent in El Tor biotype from the 7th pandemic and the Latin American epidemic strains (Olsvik et al., 1993). *V. cholerae* O1 El Tor isolates that produces classical CT is a newly emerged trait, which is said to be associated with the severity of the illness (Siddique et al., 2010) with a large number of cholera outbreaks (Nair et al., 2006; Safa et al., 2008; Raychoudhuri et al., 2009). CT encoding genes of O1 and O139 serogroups is carried by a filamentous CTXΦ, which is known to use the toxin-coregulated pili (TCP) as its receptor (Waldor and Mekalanos, 1996). *V. cholerae* O139 harboring CTX^class^Φ and CTX^calc^Φ has been described based on the difference in the sequence of *rstR* that encodes for the repressor protein of the CTXΦ (Faruque et al., 2003a; Bhattacharya et al., 2006; Raychoudhuri et al., 2010).

This study was undertaken to understand the phenotype and genetic changes of *V. cholerae* O139 isolated from sporadic hospitalized cholera cases in Delhi during 2001–2006. The outcome of this study may be useful to comprehend the epidemiology of *V. cholerae* O139.

## Materials and methods

### Bacterial strains

*V. cholerae* O139 was isolated from cholera patients admitted at the Maharishi Valmiki Infectious Diseases Hospital, Delhi. Between 2001 and 2006, 42 isolates individually isolated strains were included in this study (Table 1). *V. cholerae* O1 569B (classical biotype), N16961 (El Tor biotype), and SG-24 (serogroup O139) were used as reference strains. In the pulsed-field gel electrophoresis (PFGE), *Salmonella enterica* serotype Braenderup strain H9812 was used as the molecular size standard.

**Table 1:** **Phenotypic and genetic characteristics of *V. cholerae* O139 isolates**.

**Isolate**	**Year**	***rstR* classical**	***rstR* El Tor**	***rstR* cal**	ctxB^*^ El Tor	ctxB^*^ classical	**Ribotype**	***ctx* copy**	**Antibiogram**	**MIC (μg/ml)**					
										**A**	**Na**	**S**	**Co**	**C**	**E**
2	2001	−	+	−	−	+	BI	1	ACoS	3	−	64	4	−	−
4	2001	−	+	−	−	+	BI	1	ACoFzS	4	−	128	4	−	−
21	2001	−	+	−	−	+	BII	1	Fz	−	−	−	−	−	−
36	2001	−	+	−	−	+	BI	1	CCoS	−	−	64	12	2	−
37	2001	+	+	+	+	+	BVIII	ND	AFzNaS	3	>256	128	−	−	−
46	2001	+	+	+	+	+	BVIII	2 + 3 TRN	AFzNaSE	4	>256	>256	−	−	1.5
103	2001	+	+	+	+	+	BVIII	ND	NaS	−	>256	64	−	−	−
174	2001	−	+	−	−	+	BII	1	ACFzS	3	−	64	−	4	−
262	2001	−	+	−	−	+	BII	1	AFzS	4	−	128	−	−	−
274	2001	−	+	−	−	+	BI	1	ACFzS	4	−	>256	−	4	−
280	2001	−	+	−	−	+	BII	ND	AFzS	3	−	>256	−	−	−
X	2001	−	+	+	+	+	BII	ND	ACCoFzNaS	3	16	64	4	4	−
3653	2004	−	+	+	−	+	BII	2 + 1TRN	AFzNa	4	>256	−	−	−	−
3686	2004	−	+	+	−	+	BII	2 + 1TRN	AFzNa	6	>256	−	−	−	−
3705	2004	−	+	+	−	+	BII	2 + 1TRN	AFzNa	6	16	−	−	−	−
3710	2004	−	+	+	−	+	BII	2 + 1TRN	AFzNa	4	>256	−	−	−	−
3711	2004	−	+	+	−	+	BII	2 + 1TRN	AFzNaE	12	16	−	−	−	1
3712	2004	−	+	+	−	+	BII	2 + 1TRN	AFzNa	6	>256	−	−	−	−
3719	2004	−	+	+	−	+	BII	2 + 1TRN	FzNa	−	16	−	−	−	−
3722	2004	−	+	+	−	+	BII	2 + 1TRN	AFzNa	4	16	−	−	−	−
3736	2004	−	+	+	−	+	BII	2 + 1TRN	AFzNa	6	16	−	−	−	−
3784	2004	−	+	+	−	+	BII	2 + 1TRN	AFzNa	4	>256	−	−	−	−
3786	2004	−	+	+	−	+	BII	ND	AFzNa	6	>256	−	−	−	−
3791	2004	−	+	+	−	+	BII	ND	AFzNa	4	16	−	−	−	−
3795	2004	−	+	+	−	+	BII	2 + 1TRN	AFzNa	4	12	−	−	−	−
3796	2004	−	+	+	−	+	BII	2 + 1TRN	ANa	4	>256	−	−	−	−
3799	2004	−	+	+	−	+	BII	2 + 1TRN	AFzNa	4	16	−	−	−	−
3822	2004	−	+	+	−	+	BII	ND	FzNa	−	16	−	−	−	−
3848	2004	−	+	+	−	+	BII	2 + 1TRN	AFzNa	4	8	−	−	−	−
8/15	2004	−	+	+	−	+	BII	ND	NaE	−	16	−	−	−	1.5
24/6	2004	−	+	+	−	+	BII	ND	ANa	4	>256	−	−	−	−
12/17	2004	−	+	−	−	+	BII	ND	A	4	−	−	−	−	−
OS-227	2004	−	+	+	−	+	BII	2 + 1TRN	FzNa	−	64	−	−	−	−
5037/05	2005	−	+	+	−	+	BII	2 + 1TRN	AFzNa	4	24	−	−	−	−
130/06	2006	−	+	+	−	+	BII	2 + 1TRN	FzNa	−	16	−	−	−	−
4602/06	2006	−	+	+	−	+	BII	2 + 1TRN	AFzNa	6	>256	−	−	−	−
5340/06	2006	−	+	+	−	+	BII	2 + 1TRN	AFzNa	8	>256	−	−	−	−
5801/06	2006	−	+	+	−	+	BII	2 + 1TRN	AFzNa	6	24	−	−	−	−
5932/06	2006	−	+	+	−	+	BII	2 + 1TRN	AFzNa	4	>256	−	−	−	−
6080/06	2006	−	+	+	−	+	BII	2 + 1TRN	AFzNa	6	16	−	−	−	−
6120/06	2006	−	+	+	−	+	BII	2 + 1TRN	AFzNa	3	64	−	−	−	−
6127/06	2006	−	+	+	−	+	BI	2 + 1TRN	AFzNa	4	3	−	−	−	−

* *As identified by MAMA-PCR. Abbreviations; ND, not done; TRN, truncated gene; A, ampicillin; C, chloramphenicol; Co, co-trimoxazole; Fz, furazolidone; E, erythromycin, Na, nalidixic acid; S, streptomycin*.

*All the isolates had ICE. floR, str, and dfr genes are present in the respective chloramphenicol, streptomycin, co-trimoxazole resistant V. cholerae O139 isolates of 2001*.

### Bacteriology and serotyping

*V. cholerae* isolates were grown on thiosulphate-citrate-bile salt-sucrose (TCBS) agar (Eiken, Tokyo, Japan) at 37°C for 16–18 h. Typical sucrose fermenting yellow colonies was further streaked on Luria agar (LA, Difco, Detroit, MD, USA) and subsequently used in the rapid biochemical identification (Nair et al., 1987). Presumptively identified *V. cholerae* isolates were further confirmed by oxidase test and confirmed serologically by slide agglutination test using O1 and O139 monoclonal antibodies prepared at the National Institute of Cholera and Enteric Diseases, Kolkata, India (Garg et al., 1994; Ramamurthy et al., 1995).

### Antimicrobial susceptibility

Antimicrobial susceptibility testing was performed using commercially available disks (Difco) following the Clinical and Laboratory Standard Institute guidelines (CLSI, 2014). The concentration of antibiotics in the disc was as follows: ampicillin (10 μg), chloramphenicol (30 μg), co-trimoxazole (sulfamethoxazole/trimethoprim, 1.25/23.45 μg), ciprofloxacin (5 μg), furazolidone (100 μg), norfloxacin (10 μg), gentamycin (10 μg), nalidixic acid (30 μg), neomycin (30 μg), streptomycin (10 μg), tetracycline (30 μg), and erythromycin (15 μg). Except for furazolidone, the minimal inhibitory concentrations (MICs) of antibiotics (ampicillin, chloramphenicol, erythromycin, nalidixic acid, streptomycin, sulfamethoxazole/trimethoprim) were determined by *E*-test (AB bioMérieux, Solna, Sweden).

### Extraction of chromosomal DNA

Modified method of Murray and Thompson (1980) was used for *V. cholerae* genomic DNA extraction.

### Polymerase chain reaction (PCR) assay

Multiplex PCRs were used for the detection of *rfb* genes encoding the somatic antigen of O139/O1, CT encoding gene (*ctxA*), and biotypes based on the allelic difference in the *tcpA* gene (Keasler and Hall, 1993; Hoshino et al., 1998). Simplex PCR assays with specific primers were made for the detection of *rstR* alleles (Bhattacharya et al., 2006). MAMA-PCR was made to detect the presence of *ctxB* alleles (CT genotypes) as described previously (Morita et al., 2008). Location of CTX prophage in chromosome II was confirmed by PCR using published methods (Maiti et al., 2006). To confirm the presence of integrative conjugative element (ICE) that carries the SXT element, two sets of primers were used in this study. Primers 10SF13 (5′-TTGTGGTGGAAA GAGGGTG-3′), SXT-13 (5′-CCAACAAAGAAC AGTTTGACTC-3′), and ORF-16 (5′-CATCTACCA CTTCATAGGCAGG-3′), YND-2 (5′-CAGCTTAAC TCACCAAGGAC-3′) were designed using conserved right and left terminal ends of the ICE, respectively. In addition, *floR, str*, and *dfr* genes encoding chloramphenicol, streptomycin, co-trimoxazole resistance was identified using published methods (Hochhut et al., 2001). In these PCRs, *V. cholerae* 569B, N16961, and SG-24 were used as reference strains. PCR assays were performed using an automated thermocycler (Gene Amp PCR system 9700, Applied Biosystems, Foster City, CA).

### DNA sequencing

The 460 bp region of *ctxB* gene was amplified by PCR from eight representative isolates of *V. cholerae* O139 covering all the years (Olsvik et al., 1993). The amplified product was purified using a PCR purification kit (Qiagen, Hilden, Germany) and used directly as a template for nucleotide sequencing. Both the strands of DNA were sequenced with BigDye terminator cycle sequencing kit using an automated sequencer ABI 3700 (Applied Biosystems). The nucleotide and amino acid sequences were compared with the sequences available in the GenBank. The nucleotide sequence data generated with five representative isolates of *V. cholerae* O139 were submitted to the GenBank with accession numbers from GQ892075 to GQ892079.

### Ribotyping

A 7.5-kb *BamH*1 (Fermentas, Waltham, MA, USA) fragment of plasmid pKK3535 containing the 16S and 23S rRNA genes of *Escherichia coli* was used as a rRNA probe (Brosius et al., 1981). Standard *V. cholerae* ribotyping was followed in this study (Faruque et al., 2000). Instead of radioisotope, we used chemiluminescent dye (Gene Images Alkaphos direct labeling and detection system, Amersham Biosciences, UK) in the DNA hybridization analysis.

### *ctxA* RFLP

Restriction enzymes *HindI*II, *Pst*I, and *Bgl*II (Fermentas) were used for the digestion of *V. cholerae* O139 chromosomal DNA and immobilized on nylon membranes (Amersham International). The CT encoding gene (*ctxA*) probe was a 540-bp *Xba*I-*Cla*I (Fermentas) fragment cloned into the plasmid pKTN901 using *EcoR*1 linkers (Kaper et al., 1988). The 267-bp *cep* probe was derived from *EcoR*1 (Fermentas) digested pSC01 plasmid.

### Pulsed-field gel electrophoresis (PFGE)

PFGE of *V. cholerae* O139 was performed as described previously for *V. cholerae* O1 (Cooper et al., 2006). PFGE profiles were analyzed using the BioNumerics version 4.0 software (Applied Maths, Sint Martens Latem, Belgium). The tagged image file formats were normalized by using the universal *S. enterica* serotype Braenderup (H9812) size standard on each gel against the reference in the database. In the dendrogram analysis, the PFGE profiles were matched using the Dice coefficient and unweighted pair group method using arithmetic averages (UPGMA). Clustering of PFGE profiles was made using 1.5% band position tolerance window and 1.5% optimization.

## Results

### Identification

Conventional serology and multiplex PCRs employed in this study confirmed all the isolates as *V. cholerae* O139.

### Antimicrobial susceptibility

In the antimicrobial susceptibility testing by disc diffusion assay, more than 60% of the *V. cholerae* O139 isolates were resistant to ampicillin, furazolidone, and nalidixic acid displaying the antibiogram as AFzNa (Table 1). The susceptibility pattern of *V. cholerae* O139 isolated during 2001 differed from the rest of the study period by displaying resistance to chloramphenicol, co-trimoxazole, and streptomycin. During the same year, 66% of the isolates were susceptible to nalidixic acid. However, in the subsequent years (2004–2006), all most all the isolates were resistant to ampicillin, furazolidone, and nalidixic acid (Table 1). For neomycin, 23 isolates showed reduced susceptibility and 19 remained susceptible (data not shown). The MIC values varied considerably for ampicillin (4–12 μg/ml), co-trimoxazole (4–12 μg/ml), nalidixic acid (3 to >256 μg/ml), and streptomycin (64 to >256 μg/ml). MIC for chloramphenicol (2–4 μg/ml) and erythromycin (1–1.5 μg/ml) remained low (Table 1).

### Analysis of virulence loci, ICE and antimicrobial resistance encoding genes

The O139 isolates uniformly harbored *ctxA* with an El Tor allele of *tcpA*. In the MAMA-PCR, all the isolates were identified as CT genotype 1. In addition, four isolates (37, 46, 103, and X) collected in 2001 exhibited CT genotype 3 (Table 1). The amplified *ctxB* gene from eight isolates was directly sequenced. The deduced amino acid sequence analysis identified heterogeneity in the B subunit of CT. Some of the 2004 and 2005 isolates had aspartic acid (D), histidine (H), phenylalanine (F), and threonine (T) at positions 28, 39, 46, and 68, respectively in the CtxB, which is similar to the CT genotype 1 of the *V. cholerae* O1 classical 569B strain (Table 2). However, the isolates representing 2001, 2004, and 2006 had amino acids alanine (A), H, F, T at positions 28, 39, 46, 68, respectively, which has been classified as CT genotype 4. This genotype was described in our previous report as genotype 5 with *V. cholerae* O139 isolates from Bangladesh (Bhuiyan et al., 2009). Subsequently, this was corrected in our publication in 2010 (Raychoudhuri et al., 2010).

**Table 2:** **CT genotypes of *V. cholerae* O139 based on DNA sequences of *ctxB***.

***V. cholerae* [Isolate No.] (year of isolation)**	**No. of isolates**	**Nucleotide at position**				**Amino acid at position**				**CT genotype**
		**83**	**115**	**138**	**203**	**28**	**39**	**46**	**68**	
*V. cholerae* O1 [569B], classical	1	A	C	T	C	D	H	F	T	1
*V. cholerae* O1 El Tor, [N16961]	1	A	T	T	T	D	Y	F	I	3
*V. cholerae* O139 [37, 46, 103] (2001)	3	C	C	T	C	A	H	F	T	4^*^
*V. cholerae* O139 [3722] (2004)	1	C	C	T	C	A	H	F	T	4^*^
*V. cholerae* O139 [3705] (2004)	1	A	C	T	C	D	H	F	T	1
*V. cholerae* O139 [5037] (2005)	1	A	C	T	C	D	H	F	T	1
*V. cholerae* O139 [6080, 6127] (2006)	2	C	C	T	C	A	H	F	T	4^*^

* *New CT genotype*.

About 80% of the isolates possessed more than one allele of *rstR*, one being the El Tor type (*rstR*^*ET*^) and the other with *rstR*^*calc*^ type. Interestingly, three 2001 isolates (37, 46, and 103) carried all the three *rstR* alleles, i.e., *rstR*^*Cl*^, *rstR*^*ET*^, and *rstR*^*Calc*^. These isolates belonged to a new ribotype (Table 1). ICE was present in all the *V. cholerae* O139 isolates as confirmed by two sets of primers. *V. cholerae* O139 isolated in 2001 that were resistant to chloramphenicol, streptomycin and co-trimoxazole respectively harbored *floR, str*, and *dfr* genes.

### *ctxA* RFLP

**Figure 1:**
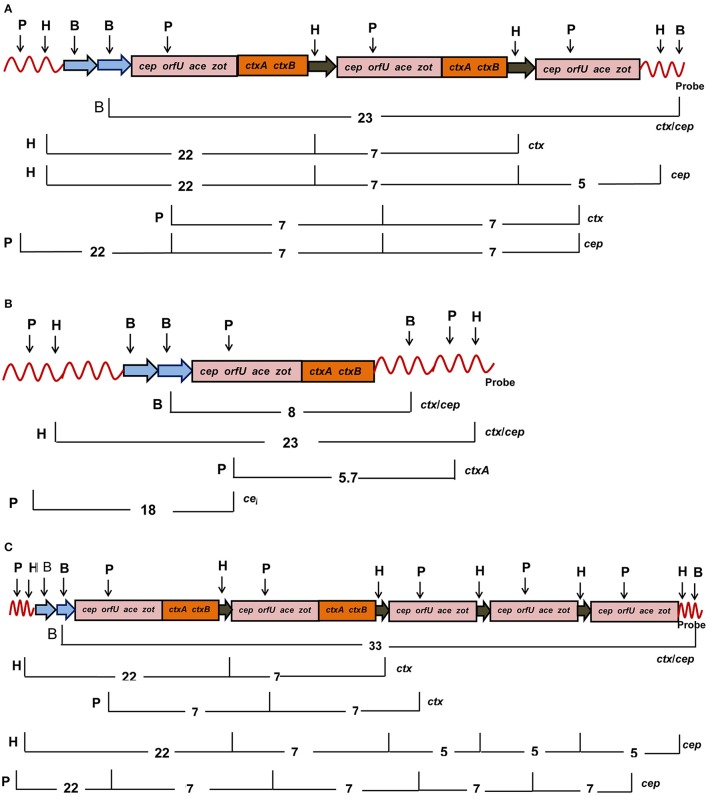
**Mapping of CTX genetic element consisting of *cep, orfU, ace, zot*, and *ctxAB* genes in *V. cholerae* O139 after digestion of chromosomal DNA with *Hind*III (H), *Pst*I (P), *Bgl*II (B), and probed with *ctxA*. (A)** Eighteen *V. cholerae* O139 isolates had two intact copies (pink and brown) and one truncated CTX elements (pink). **(B)** Six *V. cholerae* O139 isolates harbored single intact copy of the CTX element (pink and brown). **(C)** The isolate 46 had two intact copies (pink and brown) and three copies of the truncated (pink) CTX elements. Restriction sites are abbreviated as B, H, and P for *Bgl*II*, Hind*III, and *Pst*I, respectively. List of probes used for hybridization was given at the right hand side. The number denotes length of the DNA fragments in Kb after digestion with the marked restriction enzymes on the left hand side.

Twenty four *V. cholerae* O139 isolated during 2004–2006 displayed two tandemly arranged copies of intact CTX prophages with *cep, orfU, ace, zot*, and *ctxAB* as a 23 Kb fragment (Figure 1A). These CTX prophages were closely bordered with a 5 Kb truncated prophage (without *ctxAB*) as detected by the *cep* probe (Table 1, Figure 1A). Seven *V. cholerae* O139 isolated in 2001 had a single copy of CTX prophage as detected by 8 Kb *ctx/cep* probes (Table 1, Figure 1B). One isolate harbored two entire copies of CTX prophages as detected by *ctx* probe along with 3 truncated phages that were detected as three 5 Kb fragments by *cep* probe (Table 1, Figure 1C). Mapping could not be accomplished for 10 isolates with the applied strategy in this study.

### Chromosomal location of CTX prophages

Three of the 2001 isolates (37, 46, and 103) carried CTX prophages on both the chromosomes, which were confirmed by PCR with specific primers for chromosome I and II of *V*. *cholerae* (Maiti et al., 2006). In the rest of the *V. cholerae* O139 isolates, the CTX prophages remained in chromosome 1. To our knowledge, this is the first report indicating the presence of CTX prophages on chromosome II in *V. cholerae* O139.

### Ribotyping

**Figure 2:**
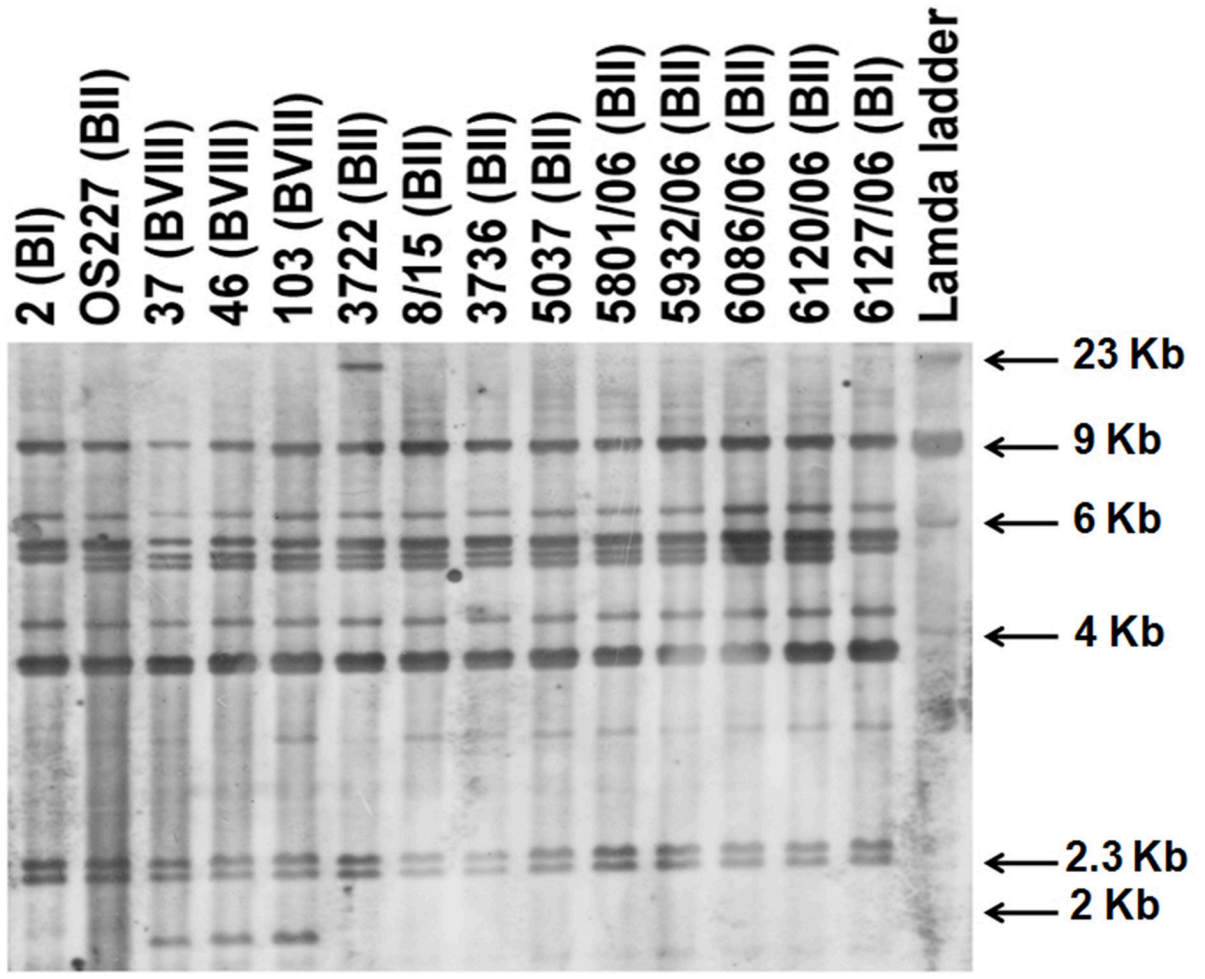
**Ribotyping of *V. cholerae* O139 isolates after digestion of chromosomal DNA with *Bgl*I**. Letter in parentheses against each isolate indicate the ribotype number.

*V. cholerae* O139 isolates exhibited three different ribotypes (Table 1, Figure 2). Ribotype BII was predominant in 34 isolates, while 5 isolates exhibited BI ribotype. All the isolates of 2004–2005 exhibited ribiotype BII pattern (Table 1). Ribotype patterns of 2001 isolates had mixture of BI (with 4 isolates) and BII (with 5 isolates). Interestingly, three isolates (37, 46, and 103) identified in 2001 exhibited a new ribotype pattern (Table 1, Figure 2). These three isolates had an extra DNA band around the 2-Kb region (Figure 2). This could be the new ribotype BVIII of *V. cholerae* O139.

### Pulsed-field gel electrophoresis (PFGE)

**Figure 3:**
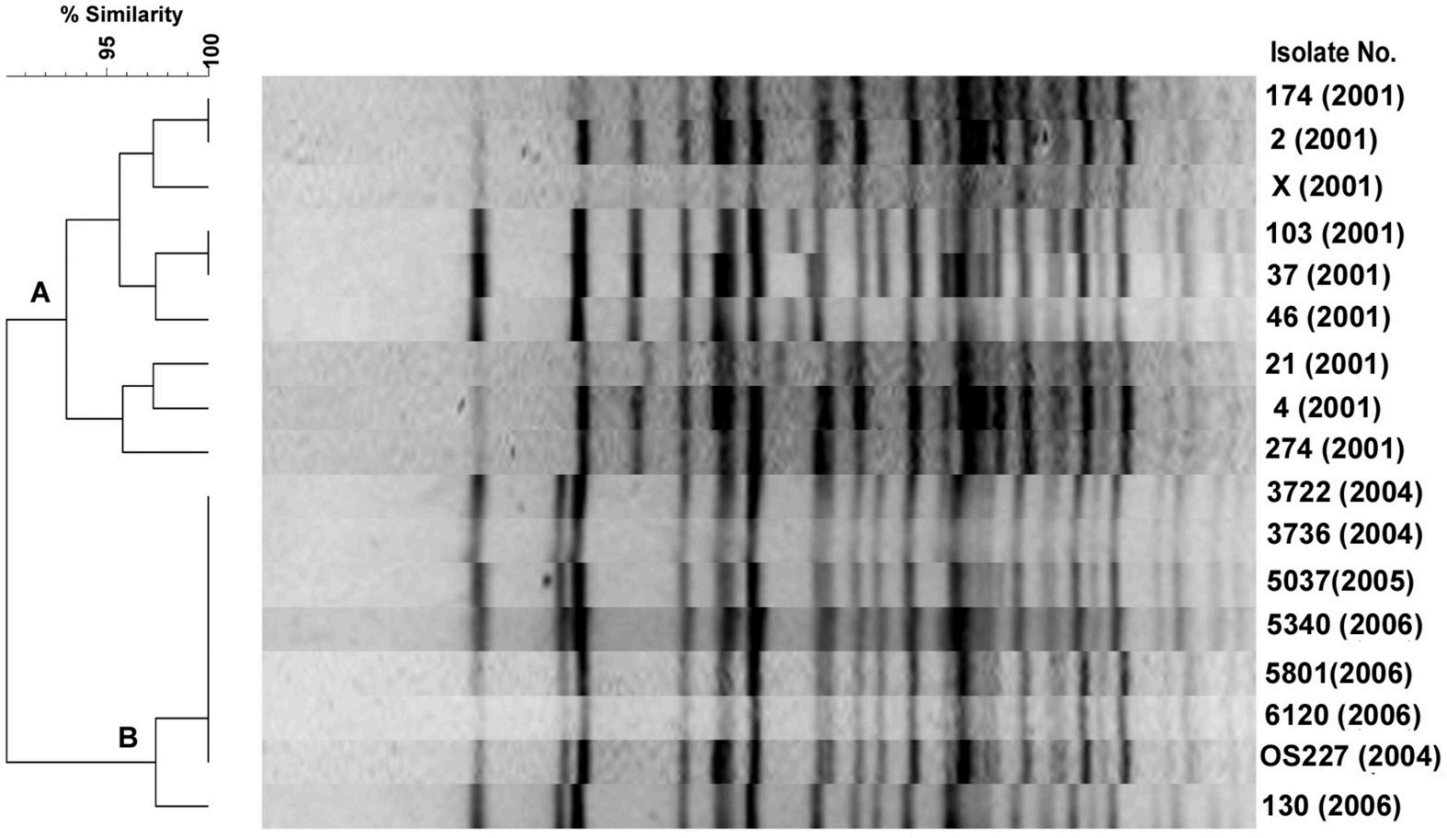
**PFGE of *V. cholerae* O139 isolates after digestion of chromosomal DNA with *Not*I**. The TIFF image of the gel was analyzed using Bionumeric software to generate the dendrogram. Percent similarity was shown at the left hand side.

Among the 9 2001 isolates, 8 different PFGE profiles were identified demonstrating the diversity of their genomes (Figure 3, cluster A). However, 3 isolates of 2001 belongs to ribotype BVIII were closely related in the PFGE. *V. cholerae* O139 isolated during 2004–2006 had similar PFGE profiles (Figure 3, cluster B), but diverged from the other isolates of 2001. A consistent correlation existed in both ribotyping and PFGE methods as most of the isolates having BII ribotype pattern were placed in clusters B. In addition, the dendrogram displayed subtypes among *V. cholerae* O139 isolates with the BII and BVIII ribotypes at about 97% similarity level (Figure 3).

## Discussion

One of the phenotypic markers used in the epidemiology of cholera is the antimicrobial susceptibility patterns. In this study, *V. cholerae* O139 isolates were resistant to ampicillin, furazolidone, and nalidixic acid, a trend observed in majority of the *V. cholerae* O1 serotype Inaba isolated during 2004–2005 from different parts of India (Dutta et al., 2006). The O139 isolates identified in 1992 were resistant to chloramphenicol, co-trimoxazole, and streptomycin (Mukhopadhyay et al., 1998). The reemerged *V. cholerae* O139 during 1996–1997 in India and Bangladesh showed susceptibility toward co-trimoxazole (Mitra et al., 1998; Faruque et al., 2003a).

In *V. cholerae* O1 and O139, mobile ICE that carried antimicrobial resistance genes in the variable region expressed resistance to chloramphenicol, co-trimoxazole, and streptomycin (Hochhut et al., 2001). In this study, ICE was detected in all the O139 isolates. However, only some of the 2001 isolates were resistant to chloramphenicol, streptomycin, and co-trimoxazole and harbored *floR, str*, and *dfr*. These resistance-encoding genes were not present in other isolates in the ICE variable region. Early studies conducted during the emergence of *V. cholerae* O139 in India showed a trend of resistance to neomycin (Mukhopadhyay et al., 1998). In this study, the O139 isolates were either susceptible or showed reduced susceptibility to neomycin. As seen in previous reports, all the *V. cholerae* O139 isolates remained susceptible to norfloxacin, tetracycline, and ciprofloxacin, which are used in the treatment of cholera (Basu et al., 2000).

The CT genotype of *V. cholerae* O1 El Tor isolates from many countries has changed from CT genotype 3 to 1 (Safa et al., 2008; Raychoudhuri et al., 2009) and such changes were detected in strains associated with large cholera outbreaks in India and Bangladesh (Kumar et al., 2009; Nguyen et al., 2009; Taneja et al., 2009). CT genotype 4 has closest homology to CT genotype 1 with a difference of only single nucleotide (nucleotide cytosine instead of adenine) at position 83 (Raychoudhuri et al., 2010). Overall, our finding matches with the observation made in *V. cholerae* O139 isolated during 1998, 2000, and 2002 from Bangladesh and Kolkata, respectively (Bhuiyan et al., 2009; Raychoudhuri et al., 2010). Compared to El Tor, the hybrid isolates with CT genotype 4 have caused larger cholera outbreaks with more severe clinical symptoms (Kumar et al., 2009; Nguyen et al., 2009; Taneja et al., 2009; Siddique et al., 2010).

Epidemiologically, the CTXΦ appear to be very important as they show the genetic changes among *V. cholerae* O1/O139 that emerged during different periods (Faruque et al., 2000; Qu et al., 2003). In the *ctxA* RFLP analysis, three prophages were encountered in different years. The unusual genetic features of the three 2001 isolates of *V. cholerae* O139 includes identification of the new ribotype BVIII pattern, the presence of three *rstR* allele types, CTX prophages of the classical type, and integration of CTX prophage in both the chromosomes. Epidemiologically, the new ribotypes of *V. cholerae* O1/O139 has been identified along with changes in the CTX prophage or *rstR* allele (Faruque et al., 1997). Considering several genetic events in the past, it has been inferred that the *V. cholerae* O139 may have multiple origins with different progenitors (Faruque et al., 2003b; Garg et al., 2003; Qu et al., 2003).

Genesis of *V. cholerae* O1 El Tor from the classical biotype, the emergence of the serogroup O139, and existence of El Tor that produces classical CT suggests that the *V. cholerae* is in a continuous state of adaptability, resulting in generation of new serogroups and/or new variants of the same serogroup. Our results suggest that the genome of *V. cholerae* O139 is dynamic and has undergone several changes since its emergence in 1992. Continuous surveillance and proper monitoring of *V. cholerae* O139 are however needed to detect subtle genetic changes in the genomes and its implications in its epidemiology, pathogenesis and persistence. Future studies should focus on epigenetic studies to find answers to the question as to why the O139 serogroup has disappeared from cholera endemic regions despite several genetic changes.

## Author contributions

RG, NS, KH, GC, and GP isolated and identified the pathogens, performed phenotypic characterization and all the genetic analysis. RB, AM, SS, GN, and TR conceived the idea analyzed the data and wrote the manuscript. All authors were involved in the compilation of the report and approved the final version.

## Funding

This work supported in part by the Japan Agency for Medical Research and Development, Ministry of Education, Culture, Sports, Science and Technology, Japan, Council of Scientific and Industrial Research, and Indian Council of Medical Research, New Delhi, India.

### Conflict of interest statement

The authors declare that the research was conducted in the absence of any commercial or financial relationships that could be construed as a potential conflict of interest.
